# Cyclosporine A Induces Cardiac Developmental Toxicity in Zebrafish by Up-Regulation of Wnt Signaling and Oxidative Stress

**DOI:** 10.3389/fphar.2021.747991

**Published:** 2021-11-12

**Authors:** Mengqi Wan, Ling Huang, Jieping Liu, Fasheng Liu, Guilan Chen, Huiwen Ni, Guanghua Xiong, Xinjun Liao, Huiqiang Lu, Juhua Xiao, Qiang Tao, Zigang Cao

**Affiliations:** ^1^ Department of General Surgery, The Affiliated Children’s Hospital of Nanchang University, Nanchang, China; ^2^ Jiangxi Engineering Laboratory of Zebrafish Modeling and Drug Screening for Human Diseases, Jiangxi Key Laboratory of Developmental Biology of Organs, College of Life Sciences, Jinggangshan University, Ji’an, China; ^3^ Department of Ultrasound, Jiangxi Provincial Maternal and Child Health Hospital, Nanchang, China

**Keywords:** cyclosporine a, cardiac toxicity, oxidative stress, wnt signaling, apoptosis

## Abstract

Due to the widely application of Cyclosporine A (CsA) as an immunosuppressant in clinic, it is necessary to study its potential toxicity. Therefore, we used zebrafish as a model animal to evaluate the toxicity of CsA on embryonic development. Exposure of zebrafish embryos to CsA at concentrations of 5 mg/L, 10 mg/L, and 15 mg/L from 12 hpf to 72 hpf resulted in abnormal embryonic development, including cardiac malformation, pericardial edema, decreased heart rate, decreased blood flow velocity, deposition at yolk sac, shortened body length, and increased distance between venous sinus and arterial bulb (SV-BA). The expression of genes related to cardiac development was disordered, and the apoptotic genes were up-regulated. Oxidative stress level was up-regulated and accumulated in pericardium in a dose-dependent manner. Astaxanthin (ATX) treatment could significantly alleviate zebrafish heart defects. CsA induced up-regulation of Wnt signaling in zebrafish, and IWR-1, an inhibitor of Wnt signaling pathway, could effectively rescue the heart defects induced by CsA. Together, our study indicated that CsA induced cardiac developmental toxicity in zebrafish larvae through up-regulating oxidative stress and Wnt signaling, contributing to a more comprehensive evaluation of the safety of the drug.

## Introduction

CsA, a lipophilic cyclic polypeptide isolated from the fungus tolypocladium inflatum, is a powerful immunosuppressant. CsA inhibits the proliferation of T cells by inhibiting the activation of calcineurin (Matsuda and Koyasu, 2000; Beauchesne et al., 2007). It is widely used in the prevention of immune rejection of organ transplantation and the treatment of T cell related autoimmune diseases (Damiano et al., 2015). Although CsA has no bone marrow toxicity of other immunosuppressants, its own toxicity also hinders the research and application. The most common is that CsA causes nephrotoxicity. In addition, CsA can also cause a series of toxic and side effects such as hepatotoxicity and neurotoxicity (Thomas and Gordon, 1986), and a series of changes in the cardiovascular system such as endothelial cell injury and inhibition of angiogenesis (Woywodt et al., 2003; Nacev et al., 2011; Kim et al., 2020). At present, CsA is not only used as immunosuppressant, but also used as a cardiac protectant in clinic. For example, CsA can improve cardiac dysfunction caused by sepsis via inhibiting calcineurin (Liu et al., 2017), and inhibit the mitochondrial permeability transition pore (MPTP) in the treatment of ischemic heart disease (Hausenloy et al., 2012). Related studies have shown that CsA has cardiotoxicity (Ozkan et al., 2012), but the specific mechanism is unknown. The cardiovascular side effects of CsA greatly limit its clinical application and research.

Zebrafish, a complex organism with highly conserved organ systems and metabolic pathways, is a commonly used toxicological biological model at present. Zebrafish is small in size, economical and easy to feed. The embryo develops *in vitro*, and the main organ systems are formed at about 72 hpf (Horzmann and Freeman, 2018), which greatly shorten the cycle of drug toxicity screening and make the process more convenient. Zebrafish is particularly suitable for cardiotoxicity studies and does not rely entirely on the functional cardiovascular system as compared to embryo models in mice and chickens. Zebrafish embryos can obtain oxygen through passive diffusion, so they can survive even with severe heart defects (Stainier, 2001). The acquisition of zebrafish transgenic lines is easier than other animal models, and the transgenic fish lines labeled with fluorescent protein can more intuitively observe a series of effects of drugs on the development of zebrafish (Bambino and Chu, 2017). More importantly, the zebrafish genome sequence is highly homologous to the human genome sequence (Howe et al., 2013), so zebrafish is an ideal biological model to simulate cardiovascular, immunological, neurological and other toxic effects in an increasingly wide range of applications.

The heart is one of the earliest organs to develop in vertebrates. The development of cardiomyocyte progenitor cells and endocardial progenitor cells is the characteristic of the beginning of heart development. The pool of cardiomyocyte progenitor cells (atrium and ventricle) located in the marginal areas of both sides of the embryo migrate to the midline and fuse to form a cardiac disc structure in the midline. After a series of differentiation and torsion in 24 hpf, the S-shaped linear lumen with circulatory function is formed (Wu et al., 2020), which is divided into atrium and ventricle. At 48 hpf, the heart begins to revolve, and the right ventricle and left atrium are formed. The heart cavity expands and begins to form cardiac circulation (Bakkers, 2011). The heart, one of the most important organs to maintain the body function, carries gas and nutrients to various tissues and organs through the blood circulation. Serious heart defects can affect the growth and development of human or animal and even threaten life. Unlike mouse and chicken cardiovascular models, zebrafish embryos can obtain oxygen through passive diffusion, so they can survive for 7 days even with severe heart defects. In addition, almost all the tools available for studying the cardiovascular system in other model systems can also be used in zebrafish models (Stainier, 2001; Sarmah and Marrs, 2016). Therefore, zebrafish as an animal model to study cardiac developmental toxicity has brought great convenience to this study.

In this study, CsA was used to intervene the embryonic development of Tg (my17: GFP) transgenic zebrafish, and cardiac developmental toxicity was observed in zebrafish treated with different concentrations of CsA. The expression of genes related to heart development was disturbed, and the level of oxidative stress was increased accordingly. Astaxanillin intervention could effectively rescue CsA induced cardiotoxicity in zebrafish. In addition, CsA induced the up-regulation of Wnt signaling, and Wnt signaling inhibitors significantly reduced the cardiotoxicity. Therefore, our study showed that CsA was cardiotoxic, which was achieved by up-regulating the Wnt signaling pathway.

## Methods and Materials

### Reagents and Materials

CsA was purchased from Chengdu Deste Biotechnology Co., Ltd. (ChengDu, China) (CAS No.59865-13-3; >98% Assay), and the drug was dissolved in DMSO. Trizol reagent, reverse transcription kit and qPCR kit were purchased from Takara (DaLian, China) and Transgen Biotech (BeiJing, China) respectively. Superoxide dismutase (SOD), malondialdehyde (MDA) and reactive oxygen species (ROS) detection reagents were purchased from Nanjing Jiancheng Bioengineering Institute (Nanjing, China). IWR-1 was purchased from MedchemExpress (New Jersey,United States) (CAS: 1127442-82-3).

### Experimental Animals

Tg(my17:GFP) and Tg(kdrl:mCherry) transgenic strains, and AB strains were purchased from China Zebrafish Resource Center. The zebrafish used were kept at 28°C, 14 h of light, and 10 h of dark under constant temperature conditions. The water for culturing the zebrafish had a pH of 7.0 and a conductivity of 500 μS/cm. The live brine shrimp were fed once at 9 a.m and 2 p.m daily. On the night before spawning, the male and female fish were placed in the mating tank at a ratio of 1:1, and the embryos the next day were collected. The collected embryos were cultured with 1% methyl blue for 10 h and abnormal and dead embryos were removed under the microscop. Healthy embryos at the same developmental stage were randomly assigned to six-well plates with 20 embryos per well. The embryos were cultured in a medium containing 0.003% PTU (Sigma, United States) to inhibit the growth of pigment.

### Chemical Treatment

Healthy embryos at the same developmental stage were randomly assigned to six-well plates with 20 embryos per well. CsA was dissolved in DMSO. 5 ml of 0.003% PTU culture medium is added to each well, so that the final concentration of CsA is 5 mg/L, 10 mg/L, 15 mg/L. Zebrafish embryos were treated with CsA at concentration of 0, 5, 10 and 15 mg/L from 12 hpf to 72 hpf respectively, and cultured in an incubator at a constant temperature of 28°C. The control group was treated with DMSO only. CsA and PTU were replaced for 3 consecutive days, and the experiment was repeated three times.

The sensibility test of zebrafish embryos exposed to CsA at different stages of cardiac development was performed as previously described (Cao et al., 2020). Healthy zebrafish embryos were randomly distributed into a six-well plate, with 20 in each well. The final concentration of the CsA treatment group was 10 mg/L, 15 mg/L, and the control group was only treated with DMSO, and the time of first dosing include 0 hpf, 12 hpf, 15.5 hpf, 19 hpf, and 48 hpf, and the effects of zebrafish exposure to CsA on the morphology and function of the heart were observed at 72 hpf.

For resuced experiment, Tg (my17: GFP) was treated with 5 mg/L, 10 mg/L, 15 mg/L CsA and 0.18 mg/L astaxanthin from 12 hpf to 72 hpf, and placed in a incubator at 28°C. Drugs were changed daily. Larva were photographed with Leica M205FA. The CsA treatment group, PTU treatment group and 0.18 mg/L astaxanthin treatment group were taken as controls. Wnt signaling pathway was activated by 15 mg/L CsA and 10 nmol Wnt signaling pathway inhibitor. The 15 mg/L CsA treatment group, the PTU treatment group and the 10 nmol inhibitor treatment group were taken as controls. The results were photographed with Leica M205FA.

### Quantification of Cardiac Morphology and Function

Heart morphology and function of zebrafish in each group were recorded and analyzed. The heart rate of zebrafish at 72 hpf was calculated. The heart morphology of zebrafish at 72 hpf was photographed under fluorescence and white light by Zeiss Discovery 20 microscope. The Zesis Discovery 20 system was used to calculate the pericardial area, yolk sac area, body length (length from head to tail) and the distance from cardiac venous sinus to cardiac artery bulb (SV-BA). Each group measured 15 pieces and repeated the experiment for 3 times.

### mRNA Level Analysis

Zebrafish were treated with CsA for 72 h, 40 juveniles were taken from each group to extract total RNA, and 1 μg total RNA was used for reverse transcription (Takara). The cDNA obtained was used for qPCR experiments on the ABI Step One Plus RT-PCR system (Applied Biosystem, CA, United States), and the experiments were repeated for 3 times. The expression of cardiac related developmental genes (GATA4, Nkx2.5, vmhc, kl2a), apoptotic related genes (p53, mdm2, bax) and Wnt signaling pathway related genes (β-catenin, lef1, axin2) were analyzed, and 2^-△△Ct^ formula was used to calculate the results. Primers were obtained from Thermo Fisher.

### Histological Analysis

Embryos treated with CsA for 72 h were collected, washed 3 times with PBS, fixed overnight in 4% PFA at 4°C, embedded in paraffin, and made into 7 um sections, which were dewaxed with xylene, dehydrated with alcohol, stained with hematoxylin and eosin, and finally sealed with neutral resin. The images of zebrafish heart section were observed and collected under light microscope.

### Acridine Orange Staining

Acriridine orange (AO) is a nucleic acid dye with unique spectral properties, which can penetrate and specifically label apoptotic cells, and emit green fluorescence (Liu et al., 2021). Zebrafish embryos at 72 hpf were washed three times in embryo culture medium, treated with 4 mg/L AO and were incubated for 30 min in the dark. Then the embryo were washed three times with culture medium, anesthetized with 0.16% tricaine, and fixed in a confocal dish with 1% low solubility agarose. Images were collected using Zeiss Microscale (Discovery, V20). The experiment was repeated for 3 times.

### Analysis of Indicators Related to Oxidative Stress

60 Juvenile zebrafish at 72 hpf were collected in each group, and washed by PBS for 3 times, 5 min each time. Total protein of each group was extracted with 0.9% normal saline, and oxidative stress indicators such as superoxide dismutase (SOD) and malondialdehyde (MDA) were detected. The absorbance was measured using the SpectraMax^®^ iD3 Multi Mode. Embryos were stained with ROS in dark for 30 min at 72 h after drug treatment and pictures were taken using Zeiss Microscale (Discovery, V20).

### Statistical Analysis

The control group and different experimental groups were statistically analyzed by one-way ANOVA and *t* test. All data were expressed as mean ± standard deviation, and *p < 0.05, **p < 0.01 and ***p < 0.001 indicated that the data were statistically significant. The F values and the df (degrees of freedom) are listed in Table 1.

**TABLE 1:** The statistical data of F, degrees of freedom.

Fig	Descriptions	F Value	Df (degrees of freedom)
Figure 1	Pericardial area	259.4	59
	SV-BA distance	71.34	59
	Body length	21.67	55
	Yolk propotion	23.42	53
	Heart rate	257.6	55
Figure 2	Cardiac related developmental genes	2.751	35
Figure 3	Pericardial area at different time periods	76.550	175
	SV-BA distance at different time periods	73.805	174
Figure 4	Apoptotic related genes	36.979	26
Figure 5	SOD	143.9	11
	MDA	1017	11
Figure 6	Pericardial area	119	19
	SV-BA distance	76.5	19
	Body length	35.05	19
	Yolk propotion	16.52	19
	Heart rate	36.86	59
Figure 7	Wnt signal pathway related genes	8.608	35

## Results

### Cyclosporine A Induced Cardiac Development Defects in Zebrafish

**FIGURE 1:**
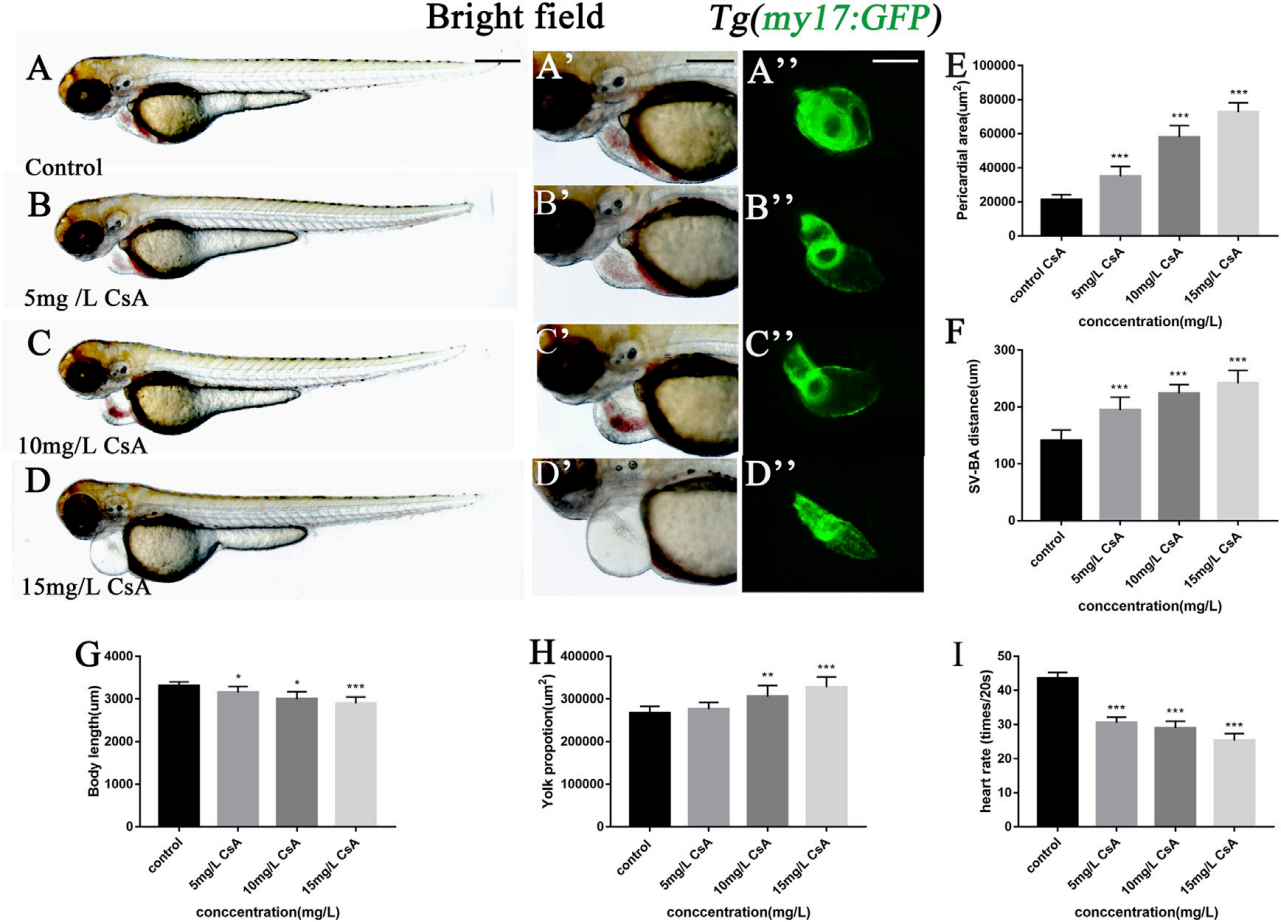
Exposure to CsA induced cardiac developmental toxicity in zebrafish embryos. **(A-D)** Tg (my17: GFP) transgenic lines were exposed to 5 mg/L, 10 mg/L, and 15 mg/L CsA from 12 hpf to 72 hpf **(E)** The pericardial area of juvenile zebrafish at 72 hpf exposed to 5 mg/L, 10 mg/L, and 15 mg/L CsA. (*n* = 15. Compared with control: *p < 0.05, ***p < 0.001, mean ± S. D). **(F)** The distance of SV-BA of juvenile zebrafish at 72 hpf exposed to 5 mg/L, 10 mg/L, and 15 mg/L CsA (*n* = 15. Compared with control:***p < 0.001, mean ± S. D). SV: sinus vein; BA: artery bulb; scale: 100 mm. **(G)** The body lenth of juvenile zebrafish at 72 hpf exposed to 5 mg/L, 10 mg/L, and 15 mg/L CsA (n = 15. Compared with control: ***p < 0.001, mean ± S. D). **(H)** Yolk sac area of zebrafish embryos at 72 hpf exposed to 5 mg/L, 10 mg/L, and 15 mg/L CsA (*n* = 15. Compared with control: *p < 0.05, mean ± S. D) **(I)** The heart rate of juvenile zebrafish at 72 hpf exposed to 5 mg/L, 10 mg/L, and 15 mg/L CsA (*n* = 15. Compared with control:***p < 0.001, mean ± SD).Scale bars:500 µm **(A-D)**, 100 µm **(A-D)**.

CsA is an immunosuppressant widely used in clinic. To study the toxic effects of CsA, zebrafish embryos were treated with different concentrations of CsA, and the heart rate, body length, yolk sac area and pericardium area of zebrafish at 72 hpf were recorded. It was found that compared with the control group (3,309 ± 92.85), embryonic body length was significantly shorter in 5 mg/L (3,153 ± 142.8,*p* < 0.001), 10 mg/L (2,998 ± 172,*p* < 0.001) and 15 mg/L (2,899 ± 146.7, *p* < 0.001) groups (Figures 1A–D,G)and blood stasis appeared in the yolk sac (Figure 1A-D). Compared with the control group, 10 mg/L and 15 mg/L groups have obvious absorption delays (Figure 1H). Heart rate was significantly lower than that of the control group, especially the 15 mg/L (25.36 ± 1.985, *p* < 0.001) group (Figure 1I). Pericardial edema worsened and was most significant at 10 mg/L (21184 ± 3,102, *p* < 0.001) and 15 mg/L (72680 ± 5,518, *p* < 0.001) (Figure 1A-D,E). The phenotypes induced by cyclosporin A including shorter body lengths, decreased heart rate, delayed absorption of yolk sac, and pericardial edema, showed obvious concentration dependence. It is worth noting that, compared with the control group, with the increase of CsA concentration, the atria and ventricles of zebrafish gradually separated, and the SV-AV distance gradually increased (Figure 1A-D,F). 5 mg/L (194.4 ± 23.05, *p* < 0.001), 10 mg/L (223.8 ± 15.86, *p* < 0.001) and 15 mg/L (241.8 ± 22.56, *p* < 0.001) have significant differences compared with the control group (140.8 ± 18.62).

**FIGURE 2:**
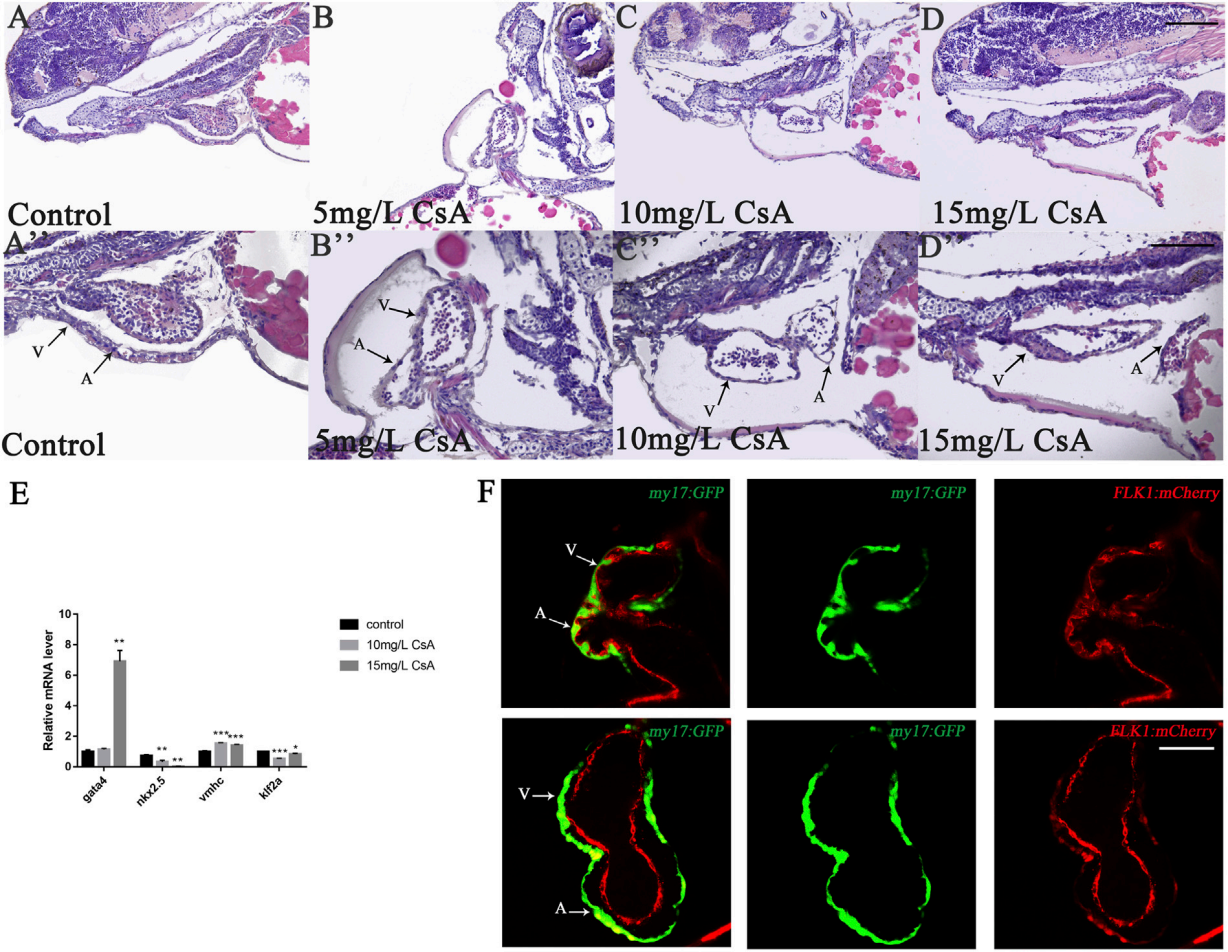
CsA exposure induced cardiac development defects. **(A-D)** HE staining of heart of zebrafish at 72 hpf exposed to 5 mg/L, 10 mg/L, and 15 mg/L CsA. Magnification: ×200 (top), ×400 (bottom); the black arrows indicated the atria and ventricles; A: atria and V: ventricles **(E)** The mRNA levels of heart-related genes in the control group and the 15 mg/L CsA exposure group (Compared with control: *p < 0.05, **p < 0.01, ***p < 0.001, mean ± S. D). **(F)** Confocal images of the cardiac region of the dual transgenic lines Tg (my17: GFP) and Tg (flk1: mCherry) in the control and treatment groups. 50 µm **(A-D)**, 25 µm **(A-D)**, 50 µm **(F)**.

The results of hematoxylin-eosin (HE) staining showed the toxic effects of CsA on cardiac development at the histological level (Figure 2A-D). In addition, the mRNA expressions of GATA4, Nkx2.5, vmhc and klf2a related to cardiac development were disturbed after CsA treatment (Figure 2E). We exposed the Tg(kdrl:mCherry) and Tg(my17:GFP) double-transgenic zebrafish embryos to 15 mg/L CsA and found that the cardiomyocytes in the drug treated group were significantly separated from the endocardia compared with the control group (Figure 2F). These results indicated that CsA had toxic effects on the heart development of zebrafish embryos.

### Cyclosporine A Initiated Cardiac Dysplasia During Precardiac Mesoderm Formation

**FIGURE 3:**
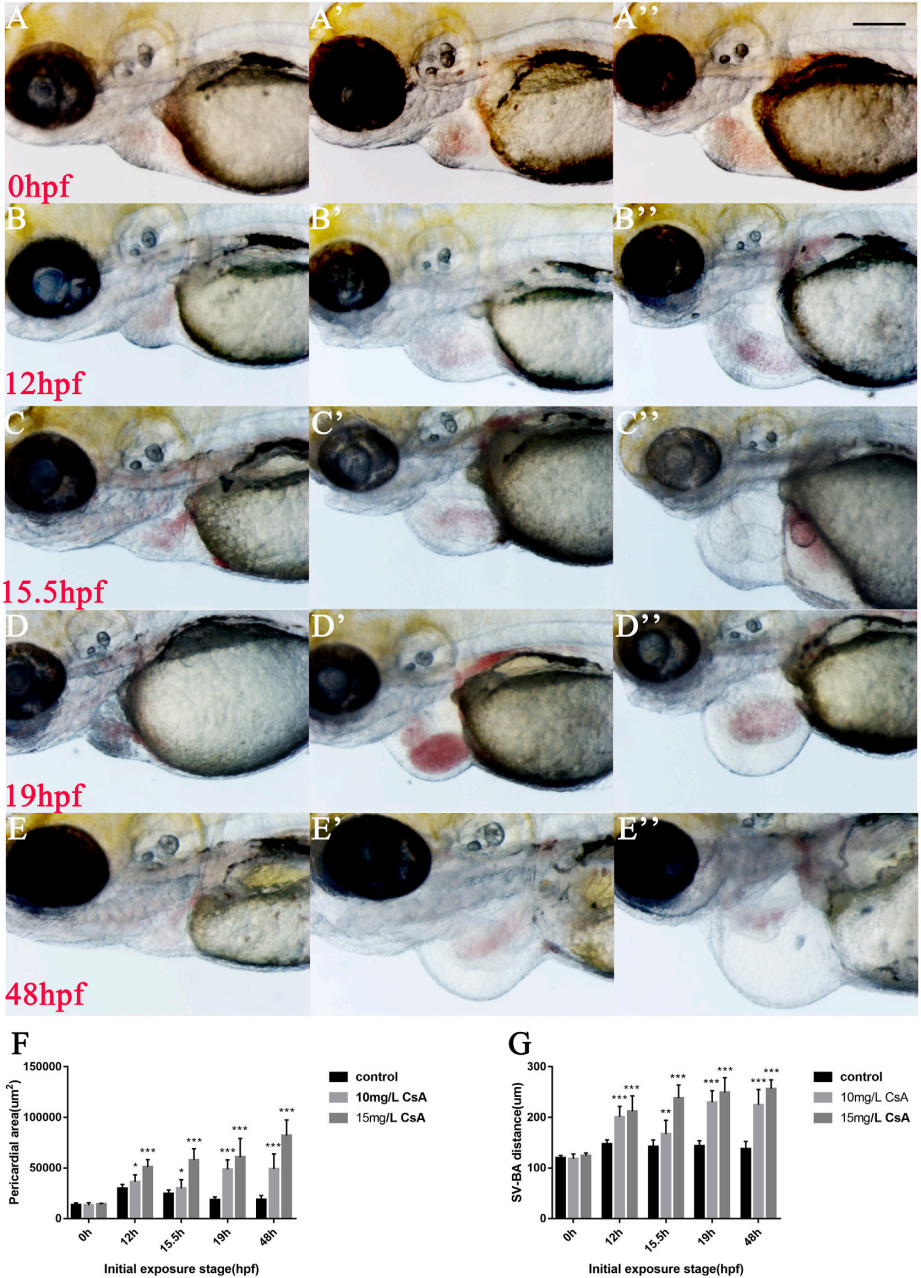
Cardiac developmental defects in zebrafish embryos induced by CsA exposure at different time periods. **(A-E)** The images of cardiac development in zebrafish embryos at 0, 12, 15.5, 19 and 48 hpf exposed to 10 mg/L and 15 mg/L CsA. **(F)** Statistical chart of pericardial area of zebrafish embryos at 0, 12, 15.5, 19 and 48 hpf exposed to CsA (Compared with control: **p < 0.01, ***p < 0.001, mean ± S. D) **(G)** Statistical chart of the distance of SV-BA of juvenile zebrafish at 0, 12, 15.5, 19 and 48 hpf exposed to CsA (Compared with control: **p < 0.01, ***p < 0.001, mean ± S. D). Scale bars:100 µm **(A-E)**.

To further investigate the role of CsA in heart development, zebrafish embryos treated with 10 mg/L and 15 mg/L in different time periods, and found that at 0 hpf, there was almost no cardiac injury in embryos (Figure 3A), while at 12 hpf, zebrafish treated with 15 mg/L CsA showed pericardial edema (Figure 3B). However, the heart injury was less severe than at other time periods. In addition, the pericardial edema increased gradually with the prolongation of treatment time, and the distance between the venous sinus and the bulb of artery also increased gradually (Figures 3A–G). These results suggested that CsA might initiate cardiac dysplasia during precardiac mesodermal formation (12 hpf).

### Cyclosporine A Induced Apoptosis of Embryonic Cardiomyocytes in Zebrafish

**FIGURE 4:**
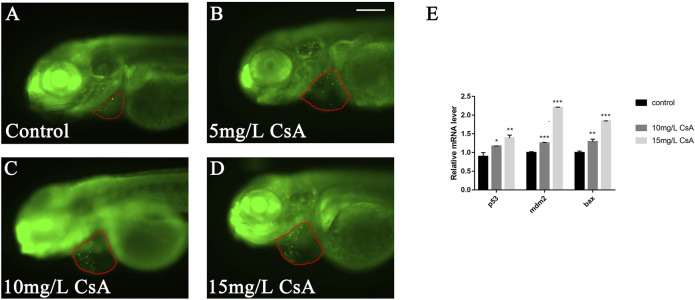
CsA-induced apoptosis of zebrafish cardiomyocytes. **(A-D)** AO staining of heart of zebrafish embryos at 72 hpf exposed to 5 mg/L, 10 mg/L, and 15 mg/L CsA. The red dotted line indicated the heart area, and the green fluorescent dots indicated apoptotic cells. **(E)** mRNA levels of apoptotic and anti-apoptotic genes in control group and 10 mg/L CsA treated group (Compared with control: *p < 0.05, **p < 0.01, ***p < 0.001, mean ± S. D). Scale bars: 100 µm **(A-D)**.

To study whether CsA induced cardiac development defects by inducing apoptosis of cardiomyocytes, we collected juvenile zebrafish treated with CsA for 72 h and stained them with AO to detect the expression of apoptosis related genes. The results showed that the number of apoptotic cardiomyocytes (green label) increased with increasing drug exposure concentration (Figures 4A–D). qPCR results showed that after CsA treatment, pro-apoptotic gene bax, p53 and mdm2 were significantly up-regulated compared with the control group (Figure 4E). These results suggested that CsA induced apoptosis of zebrafish cardiomyocytes.

### The Oxidative Stress Response Induced by Cyclosporine A Was Concentrated in the Heart Region

**FIGURE 5:**
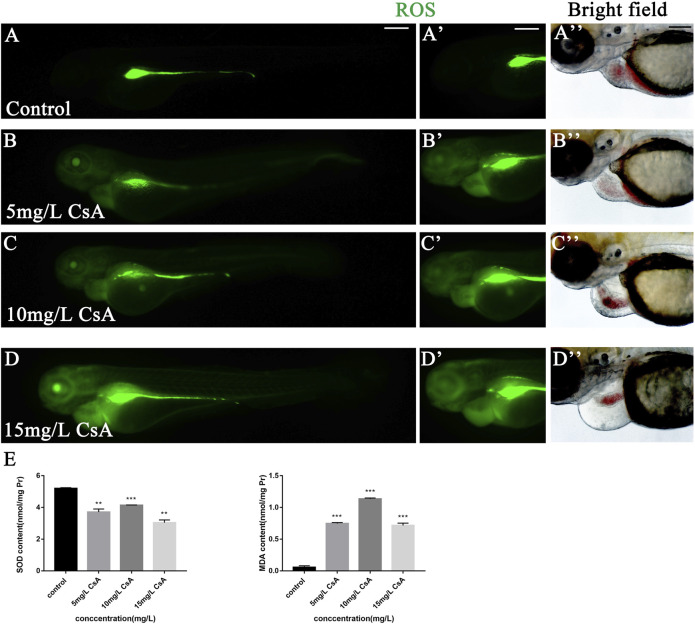
The accumulation of oxidative stress in zebrafish induced by CsA. **(A-D)** ROS staining of heart of zebrafish embryos at 72 hpf exposed to 5 mg/L, 10 mg/L, and 15 mg/L CsA. The ROS staining was green. **(E)** The SOD activity and MDA content of zebrafish at 72 hpf exposed to 5 mg/L, 10 mg/L, and 15 mg/L CsA (Compared with control: **p < 0.01, ***p < 0.001, mean ± S. D). Scale bars:100 µm **(A-D)**.

**FIGURE 6:**
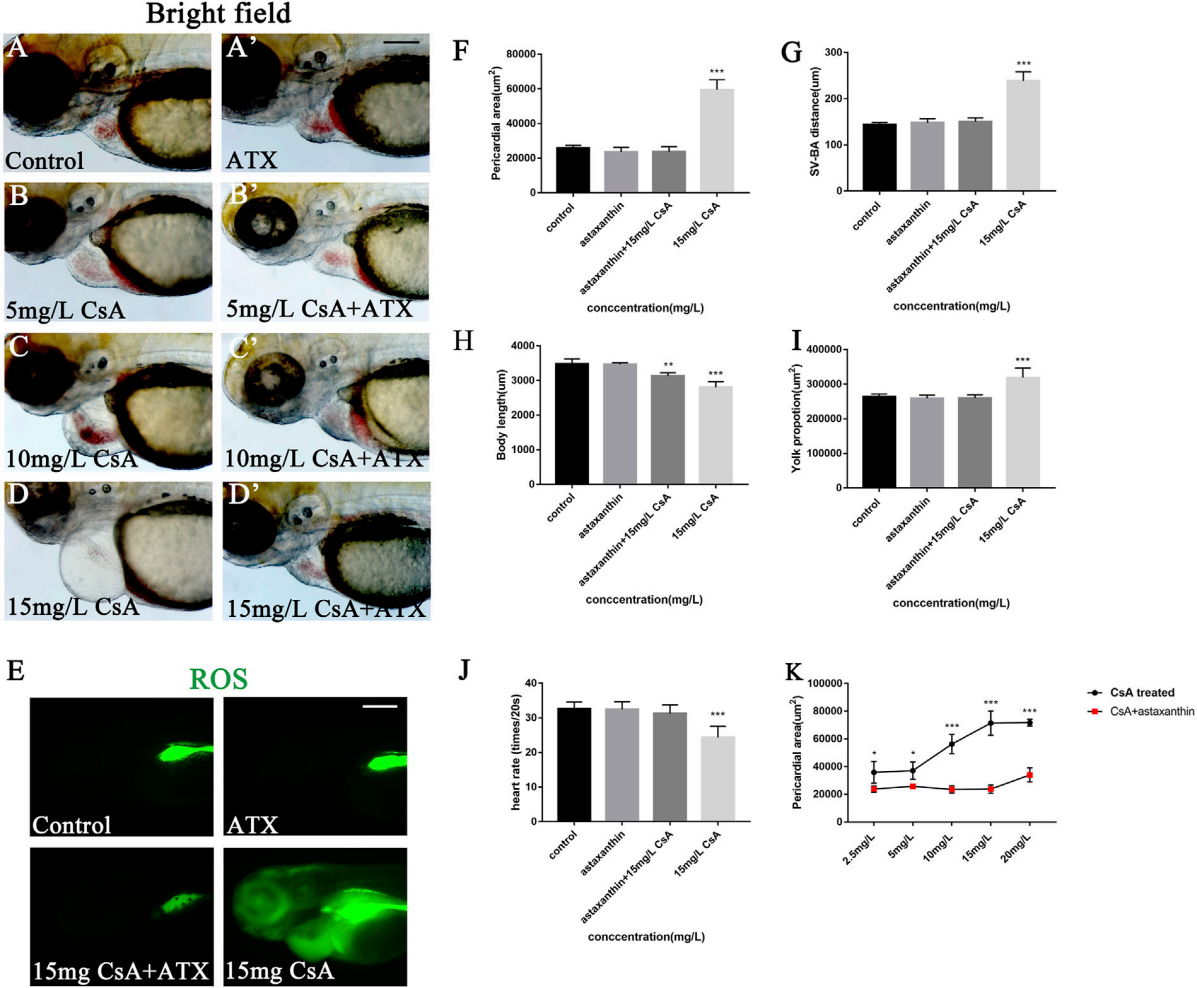
ATX rescued the cardiac developmental toxicity induced by CsA. **(A-D)** ATX rescued cardiac defects in 72 hpf zebrafish exposed to CsA at 5 mg/L, 10 mg/L, 15 mg/L. **(E)** ATX rescued ROS staining of 72 hpf zebrafish exposed to CsA. **(F)** ATX relived pericardial area of 72 hpf zebrafish exposed to CsA (*n* = 15. Compared with control: *p < 0.05, ***p < 0.001, mean ± S. D). **(G)** ATX rescued the distance of SV-BA of 72 hpf zebrafish exposed to CsA (*n* = 15. Compared with control: ***p < 0.001, mean ± S. D). SV: sinus vein; BA: artery bulb; scale: 100 mm **(H)** ATX rescued the body length of 72 hpf zebrafish exposed to CsA (*n* = 15. Compared with control: ***p < 0.001, mean ± S. D). **(I)** ATX rescued yolk sac area of 72 hpf zebrafish exposed to CsA (*n* = 15. Compared with control: *p < 0.05, mean ± S. D). **(J)** ATX rescued the heart rate of 72 hpf zebrafish exposed to CsA (*n* = 15. Compared with control: ***p < 0.001, mean ± S. D). **(K)** dose-response curve. Scale bars:100 µm **(A-H)**.

Oxidative stress plays an important role in physiological and pathological changes of all aerobic organisms (Li et al., 2016). To study the mechanism of CsA induced cardiotoxicity in zebrafish, oxidative stress level after CsA treatment was reflected by detecting ROS and MDA content, and SOD activity. The results of ROS staining showed that oxidative stress response accumulated in the heart and head of zebrafish, and the fluorescence intensity of ROS staining gradually increased with the increase of CsA concentration (Figure 5A-D). SOD activity test showed that compared to control group (5.204 ± 0.043), SOD activity in 5 mg/L (3.713 ± 0.191,*p* < 0.01), 10 mg/L (4.136 ± 0.023, *p* < 0.001) and 15 mg/L (3.041 ± 0.173, *p* < 0.01)significantly decreased. MDA content in 5 mg/L (0.747 ± 0.015,*p* < 0.001), 10 mg/L (1.135 ± 0.014, *p* < 0.001) and 15 mg/L (0.717 ± 0.036, *p* < 0.001) was significantly up-regulated compared to control group (0.056 ± 0.025, *p* < 0.001) (Figures 5E,F). The results of SOD and MDA were also consistent with the results of ROS staining (Figure 5A-D). Then we used astaxanthin (ATX) (an antioxidant) to rescue CsA-induced cardiotoxicity in zebrafish. Compared with the control group and the zebrafish without astaxanthin treatment, the heart rates of astaxanthin rescued group (31.27 ± 2.52, *p* > 0.05) have little difference from that of the control group (32.67 ± 1.915) (Figure 6J), and the pericardial edema was significantly reduced (Figure 6A-D,F). The dose-response curve showed that astaxanthin could effectively reduce pericardial edema in zebrafish (Figure 6K). The SV-AV distance had little difference between rescued group (150.3 ± 8.277,*p* > 0.05) and control group (143.5 ± 9.972,*p* > 0.05) and the symptoms of atrial and ventricular separation were reduced (Figure 6G). And the fluorescence intensity of ROS staining decreased significantly (Figure 6E). Therefore, it could be inferred that oxidative stress played a role in CsA induced cardiac dysplasia.

### Cyclosporine A Induced Abnormal Heart Development in Zebrafish by Up-Regulating Wnt Signaling

**FIGURE 7:**
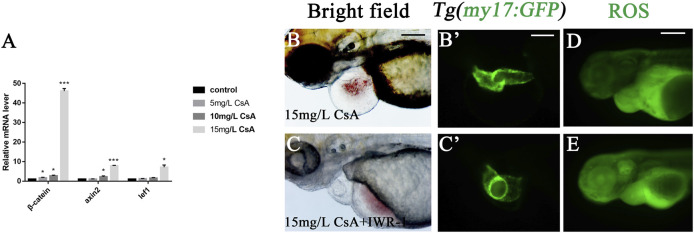
IWR-1 rescued cardiac development defects induced by CsA exposure. **(A)** The up-regulation of β-catenin, lef1 and axin2 in the heart of 72 hpf zebrafish exposed to 15 mg/L CsA (Compared with control:*p < 0.05, **p < 0.01, mean ± SD). **(B-C)** IWR-1 rescued heart defects in 72 hpf zebrafish exposed to CsA at 15 mg/L. **(D-E)** IWR-1 rescued ROS staining in 72 hpf zebrafish exposed to CsA at 15 mg/L. Scale bars:100 µm (**B-E**).

The up-regulated expression of Wnt signaling can be observed in many cardiovascular diseases, and Wnt signal plays an important role in many cardiovascular pathological changes (Foulquier et al., 2018). To investigate whether Wnt signaling is involved in CsA induced cardiac dysplasia, we detected the expression of Wnt signaling-associated genes β-catenin, lef1 and axin2, and the results showed that the expression of these three genes was significantly up-regulated after CsA treatment, especially β-catenin (Figure 7A). This suggested that the abnormal cardiac development induced by CsA might be related to Wnt signaling. To further verify this result, we treated zebrafish with IWR-1, a Wnt signaling inhibitor, and CsA. At 72 hpf, IWR-1 significantly rescued CsA-induced cardiac dysplasia compared with the zebrafish treated without IWR-1 (Figure 7B-C). The results of ROS staining in IWR-1 rescued group were not significantly different from those in 15 mg/L CsA-treated group (Figures 7D,E), suggesting that CsA-induced cardiac developmental toxicity in zebrafish might be caused by the combination of up-regulation of oxidative stress level and up-regulation of Wnt signaling in zebrafish heart.

## Discussion

Recently, zebrafish has been widely used in environmental toxicology, pathological toxicology and embryonic developmental toxicology. Many studies have confirmed that zebrafish larva have the transparency to directly evaluate drug toxicity *in vivo*, and the toxicity characteristics are similar to those of mammals, such as hepatotoxicity, cardiotoxicity and neurotoxicity (Cao et al., 2020; Wang et al., 2020; Xiong et al., 2020). CsA is an immunosuppressant widely used clinically and is also accompanied by a series of side effects such as liver toxicity, neurotoxicity and vascular toxicity. However, little is known about its effects on embryonic development Therefore, we used zebrafish to evaluate CsA toxic effects and the related mechanisms. The results showed that CsA could induce developmental toxicity and cardiotoxicity in zebrafish larvae. Further studies suggested that generation of ROS and activation of wnt signaling pathway might be the underlying mechanism of cardiotoxicity induced by CsA. However, an important question about the human relevance of the concentrations used in the zebrafish assay still need to be investigated.

To study the underlying mechanism of cardiac developmental toxicity induced by CsA, expression levels of genes correlated with cardiac development, and apoptosis were detected by qPCR. GATA4 is one of the earliest developing transcription factors during heart development, and its up-regulated expression can promote the differentiation of embryonic stem cells into heart (Grepin et al., 1997). Nkx2.5 regulates cardiac tube elongation and has different effects on the number of ventricular and atrial cells (Targoff et al., 2008). Gata4 and NKX2.5 play an important role in the differentiation, maturation and homeostasis of cardiomyocytes. The deficiency of Gata4 leads to failure of heart tube formation in mice, and the targeted destruction of Nkx2.5 leads to abnormal heart morphology (Schlesinger et al., 2011). Activation of the both is essential in stretch-induced cardiomyocyte hypertrophy (Valimaki et al., 2017). Klf2a is a major endocardial blood flow response gene, and its expression enables endocardial cells (EDCs) to coupling mechanical transduction to valve morphology by activating a series of downstream target genes (Steed et al., 2016). Ventricular myosin heavy chain (Vmhc), expressed primarily in the ventricle, can be used to distinguish between two types of cardiac precursors at an early stage before the formation of cardiac catheters (Yelon et al., 1999). Our results showed that CsA treatment led to disruption in the expression of the above heart-related transcriptional genes and upregulation of apoptotic gene bax, p53 and mdm2.

Oxidative stress is one of reasons for toxicity caused by drug. The imbalance between the production of ROS and the endogenous antioxidant defense system results in oxidative stress (van der Pol et al., 2019). Malondialdehyde (MDA) and superoxide dismutase (SOD) are important indicators of oxidative stress. MDA is a product of the peroxidation of polyunsaturated fatty acids, which can interact with DNA and proteins to induce mutations or atherosclerosis (Del Rio et al., 2005). SOD is an ubiquitous antioxidant enzyme that catalyzes the conversion of superoxide hydrogen ion radical (O2-) to hydrogen peroxide (H2O2) (Sakamoto and Imai, 2017). Excessive accumulation of ROS can lead to a variety of cardiovascular diseases, such as endothelial dysfunction and, atherosclerosis (Victor et al., 2009; Chistiakov et al., 2018), and it has been shown that the accumulation of ROS has a toxic effect on the heart development of zebrafish (Cao et al., 2020; Meng et al., 2020). Our experimental results showed that with the increase of CsA concentration, the accumulation of ROS in the pericardium of juvenile zebrafish gradually increased and SOD activity gradually decreased, while MDA content significantly increased. This suggested that CsA caused oxidative stress accumination in zebrafish. Astaxanthin (ATX) is a kind of lutein carotenoid with strong antioxidant, anti-inflammatory and anti-apoptotic activities (Brotosudarmo et al., 2020). Studies by Cun Dong Fan and his colleagues have shown that ATX can improve heart defects by eliminating reactive oxygen species (ROS) and inhibiting oxidative damage to inhibit homocysteine (Hcy)–induced cardiotoxicity (Fan et al., 2017). Here, ATX was used to interfere with CsA induced zebrafish cardiotoxicity, and the results showed that ATX had a significant inhibitory effect on CsA-induced cardiotoxicity, and the ROS level in ATX group was significantly decreased. This provided further evidence that the cardiotoxicity of CsA to zebrafish might be caused by oxidative stress.

Wnt signaling is a secreted glycoprotein that regulates cell proliferation, survival, and behavior in both vertebrates and invertebrates. Wnt/β-catenin is the most studied signaling pathway. In the absence of Wnt signaling, β-catenin is phosphorylated or degraded in the cytoplasm. When Wnt signaling is activated, β-catenin accumulates in the nucleus (Moon, 2005). The Wnt/β-catenin signaling pathway is involved in the formation of the right ventricle in zebrafish and plays an important role in the proliferation and regeneration of mature cardiomyocytes (Fan et al., 2018). Therefore, the expression of Wnt signaling-associated genes β-catenin, lef1 and axin2 was detected, and the results showed that the expression of these three genes was significantly up-regulated after CsA treatment, especially β-catenin. Wnt signal inhibitor IWR-1 could alleviate the heart defects caused by CsA, but there was no significant difference in ROS staining between the IWR-1 rescue group and the CsA-exposed group only. This suggested that zebrafish heart malformation might be induced by both the up-regulation of ROS and the activation of Wnt signaling. In conclusion, CsA can induce cardiac developmental toxicity in zebrafish larvae. Our results show that CsA exposure caused pericardial edema, body length shortened, and yolk sac absorption delayed. Moreover, CsA induced generation of ROS and apoptosis of cardiomyocytes, and activated Wnt signaling. These results indicate that CsA may induce zebrafish cardiotoxicity by generation of oxidative stress (ROS) and activation of Wnt signaling. Our findings will be helpful to understanding CsA-induced caidiac developmental toxicity and the underlying mechanism, and provide reference for new treatment and prevention methods for the side effects of clinical use of CsA and new evidence of the influence of CsA exposure on aquatic organisms.

## Data Availability

The original contributions presented in the study are included in the article/Supplementary Material, further inquiries can be directed to the corresponding authors.
